# Pericapsular nerve group (PENG) block at 10 mL vs. 20 mL for postoperative analgesia after total hip arthroplasty: a randomized controlled trial

**DOI:** 10.1186/s12871-026-03923-8

**Published:** 2026-05-18

**Authors:** Gökçe Aliş, Mesure Gül Nihan Özden, Rasim Onur Karaoğlu

**Affiliations:** 1 Sancaktepe Prof. Dr. İlhan Varank Eğitim Araştırma Hastanesi, Istanbul, Türkiye; 2 Sağlık Bakanlığı Göztepe Prof. Dr. Süleyman Yalçın Şehir Hastanesi, Istanbul, Türkiye; 3 Bağcılar Eğitim ve Araştırma Hastanesi, Istanbul, Türkiye

**Keywords:** Pericapsular nerve group block, Total hip arthroplasty, Postoperative pain, Bupivacaine, Patient-controlled analgesia

## Abstract

**Background:**

The pericapsular nerve group (PENG) block is increasingly used for analgesia in hip-related procedures. However, the optimal local anesthetic volume remains unclear. This randomized non-inferiority trial aimed to compare the analgesic efficacy of 10 mL versus 20 mL PENG block after total hip arthroplasty, hypothesizing that the lower volume would provide non-inferior analgesia.

**Methods:**

In this single-center randomized controlled non-inferiority trial, 50 patients undergoing hip replacement surgery received an ultrasound-guided PENG block with 0.25% bupivacaine, either 20 mL (*n* = 25) or 10 mL (*n* = 25). Postoperative analgesia was provided using morphine patient-controlled analgesia. Pain scores at rest and during movement were assessed at predefined time points up to 24 h postoperatively. The primary outcome was the NRS pain score at rest 1 h after surgery. Secondary outcomes included opioid consumption, quadriceps muscle strength, postoperative nausea and vomiting, and patient satisfaction.

**Results:**

There were no significant differences between groups in opioid-related side effects, quadriceps muscle weakness, patient satisfaction, or length of hospital stay. Non-inferiority analysis demonstrated that the median differences in NRS scores between groups remained within the predefined non-inferiority margin of 1 NRS point at all assessment time points. Non-inferiority of the 10 mL PENG block was confirmed for the primary outcome.

**Conclusions:**

A 10 mL PENG block provides postoperative analgesia non-inferior to a 20 mL volume without increasing opioid consumption or motor weakness, suggesting that lower volumes may be sufficient and potentially safer after total hip arthroplasty.

**Trial registration:**

ClinicalTrials.gov, NCT06166602. Registered on December 04, 2023. Retrospectively registered (study start date July 30, 2023).

**Supplementary Information:**

The online version contains supplementary material available at 10.1186/s12871-026-03923-8.

## Introduction

Hip replacement surgery is widely performed to relieve pain and restore functional capacity in patients with advanced hip joint pathology [1]. Despite improvements in surgical techniques and perioperative care, postoperative pain remains a significant clinical challenge, as inadequate analgesia may delay mobilization, prolong hospital stay, and reduce patient satisfaction [1, 2].

Postoperative pain management after hip surgery traditionally relies on systemic analgesics such as non-steroidal anti-inflammatory drugs and opioids [3]. Although effective, these medications are frequently associated with dose-dependent adverse effects, including nausea, vomiting, ileus, sedation, and respiratory depression [3]. In addition, analgesic strategies targeting a single nociceptive pathway may be insufficient to address the complex innervation of the hip joint [2, 4].

Consequently, regional anesthesia techniques have gained increasing importance in hip surgery [5]. The pericapsular nerve group (PENG) block is a relatively novel regional anesthesia technique designed to selectively anesthetize the articular branches of the femoral, obturator, and accessory obturator nerves, which play a key role in hip joint nociception [4, 6]. Previous clinical studies have shown that the PENG block provides effective postoperative analgesia while preserving quadriceps motor function, making it particularly attractive within enhanced recovery after surgery (ERAS) protocols [7–9].

Despite its growing clinical adoption, the optimal local anesthetic volume for the PENG block remains unclear. Local anesthetic volume may influence the extent of block spread, analgesic efficacy, and the risk of unintended motor blockade [10, 11]. Previous studies investigating different volumes and concentrations have reported conflicting results, and no consensus has been reached regarding the minimal effective volume that ensures adequate analgesia without increasing adverse effects [8, 10–12].

This uncertainty represents an important knowledge gap in current clinical practice. Therefore, the primary aim of this randomized controlled non-inferiority trial was to compare the analgesic efficacy of two different local anesthetic volumes (10 mL vs. 20 mL) administered during the PENG block in patients undergoing hip replacement surgery. Secondary outcomes included postoperative opioid consumption, quadriceps muscle strength, postoperative nausea and vomiting, patient satisfaction, and length of hospital stay. We hypothesized that a lower-volume PENG block would provide analgesia non-inferior to a higher volume while potentially reducing volume-related risks.

## Methods

We conducted a single-center, prospective, randomized controlled non-inferiority trial including 50 patients undergoing hip replacement surgery. The primary outcome was the Numerical Rating Scale (NRS) pain score at rest at 1 h postoperatively. A non-inferiority margin of 1 NRS point was prespecified based on IMMPACT recommendations, which consider a 1-point difference clinically meaningful in postoperative pain trials [13]. Secondary outcomes included active and rest NRS scores at predefined time points, 24-hour morphine consumption via patient-controlled analgesia (PCA), quadriceps muscle strength, postoperative nausea and vomiting (PONV), patient satisfaction, and length of hospital stay.

The study was approved by the institutional ethics committee (protocol no: 2023/0455), and written informed consent was obtained from all participants. The trial was registered at ClinicalTrials.gov (NCT06166602) and was retrospectively registered due to administrative delay, although patient enrollment and data collection strictly followed the predefined protocol.

Participants were randomly assigned to either Group I (20 mL of bupivacaine) or Group II (10 mL of bupivacaine). The age, sex, body mass index (BMI), American Society of Anesthesiologists (ASA) score and comorbidities (hypertension, diabetes mellitus, cardiovascular system disease, chronic obstructive pulmonary disease, smoking, chronic renal failure, malignancy) were recorded. Standard monitoring, including electrocardiography, peripheral oxygen saturation, and noninvasive blood pressure monitoring, was performed.

Participants were randomized 1:1 to 20 mL vs. 10 mL 0.25% bupivacaine using a computer-generated sequence (IBM SPSS Statistics for Windows, version 26.0; IBM Corp., Armonk, NY, USA) with concealed allocation (sequentially numbered, opaque, sealed envelopes). Outcome assessors and data analysts were blinded to group assignment. Patients were under general anesthesia during block administration and were therefore unaware of group allocation. PACU/ward staff involved in postoperative care and rescue analgesia administration were also blinded to group assignment.

### Inclusion criteria

Patients aged 18–80 with ASA scores of I-III who underwent hip surgery and provided informed consent were enrolled.

### Exclusion criteria

Patients with coagulopathy, chronic opioid use, substance abuse, or chronic pain syndromes requiring ongoing opioid therapy, history of LA drug allergy-toxicity, advanced organ failure, mental retardation, infection at the injection site, pregnant women and children were excluded.

### Anesthesia procedure

General anesthesia was induced with intravenous midazolam (1 mg), fentanyl (1 µg/kg), propofol (1.5–2 mg/kg), and rocuronium (0.6 mg/kg), followed by tracheal intubation. Anesthesia was maintained with sevoflurane (0.8–1 MAC) and remifentanil infusion. Ventilation was adjusted to maintain an end-tidal CO₂ of 30–35 mmHg. Intravenous morphine (0.1 mg/kg) was administered before skin closure.

### PENG block procedure and timing

The PENG block was performed in the operating room immediately after completion of surgery and before extubation. All PENG blocks were performed by a single attending anesthesiologist with 15 years of experience in ultrasound-guided regional anesthesia. While the block was applied, the psoas muscle and hip joint were visualised with Ultrasound (Samsung Ultrasound H60; Hampshire, Korea) using a linear probe. The block needle (Stimuplex^®^ A 100 mm 22 gauge, Braun Melsungen AG, Melsungen, Germany) was advanced with the in-plane technique, and 20 mL of 0.25% bupivacaine (Buvasin 0.5% vial, Vem İlaç Sanayi ve Ticaret Ltd. Şti., İstanbul -Turkey). In Group I, 20 mL of 0.25% bupivacaine was injected into the plane between the psoas tendon and the anterior hip capsule; in Group II, 10 mL of 0.25% bupivacaine was injected into the same plane. Then, patients with adequate respiratory effort below 0.2 MAC with sevoflurane were extubated after receiving intravenous administration of atropine (0.1 mg/kg) and neostigmine (0.05 mg/kg), and the duration of anaesthesia and surgery was recorded. Patients and outcome assessors were blinded to group allocation. The anesthesiologist performing the block was not blinded due to the nature of volume preparation. A standardized ultrasound-guided technique was used in all patients to minimize operator-dependent variability.

Intravenous morphine patient-controlled analgesia (PCA) was administered to the patients at a concentration of 0.5 mg/mL, a 2 mL bolus, and a 15-minute lock time. Time zero (0 h) was defined as arrival to the PACU. All postoperative assessments were recorded relative to this time point. The 1-hour NRS measurement (primary outcome) was obtained at 60 min after PACU arrival, within a predefined ± 10-minute window. The postoperative pain of the patients was recorded as pain with hip joint movement (active) and pain at rest (passive) at 15 min, 30 min, 1 h, 2 h, 4 h, 8 h, 16 h, and 24 h postoperatively. When the Numerical rating scale (NRS) value was 4 and above, tenoxicam 20 mg intravenously was administered as rescue analgesia. Tenoxicam was used as part of a standardized multimodal analgesia protocol to reduce opioid requirements and opioid-related adverse effects.

The patients’ PCA consumption and the amount of additional analgesia used were recorded for evaluation. The patients’ independent knee extension movement and quadriceps muscle strength were evaluated. Quadriceps strength was assessed using the Medical Research Council (MRC) 0–5 scale by a blinded assessor. Postoperative nausea and vomiting were recorded at 15 min, 30 min, 1 h, 2 h, 4 h, 8 h, 16 h, and 24 h. Nausea and vomiting descriptive scale (0 = None, 1 = Mild nausea, 2 = Nausea, 3 = Vomiting once and 4 = Vomiting more than once) was evaluated and recorded, while ondansetron 0.15 mg/kg was administered intravenously to patients with a scale of 2 and above. Patient satisfaction was assessed using a 5-point Likert scale (1 = very dissatisfied, 5 = very satisfied).

Side effects thought to be related to opioid use (respiratory depression, sedation, nausea, pruritus and constipation) were also recorded. In addition, it was planned to record any complications that developed due to PENG block (such as muscle weakness, LAST, nerve injury, infection and bleeding). Patients’ satisfaction was evaluated and recorded with the Likert Satisfaction Scale 24 h after the surgery.

**Fig. 1:**
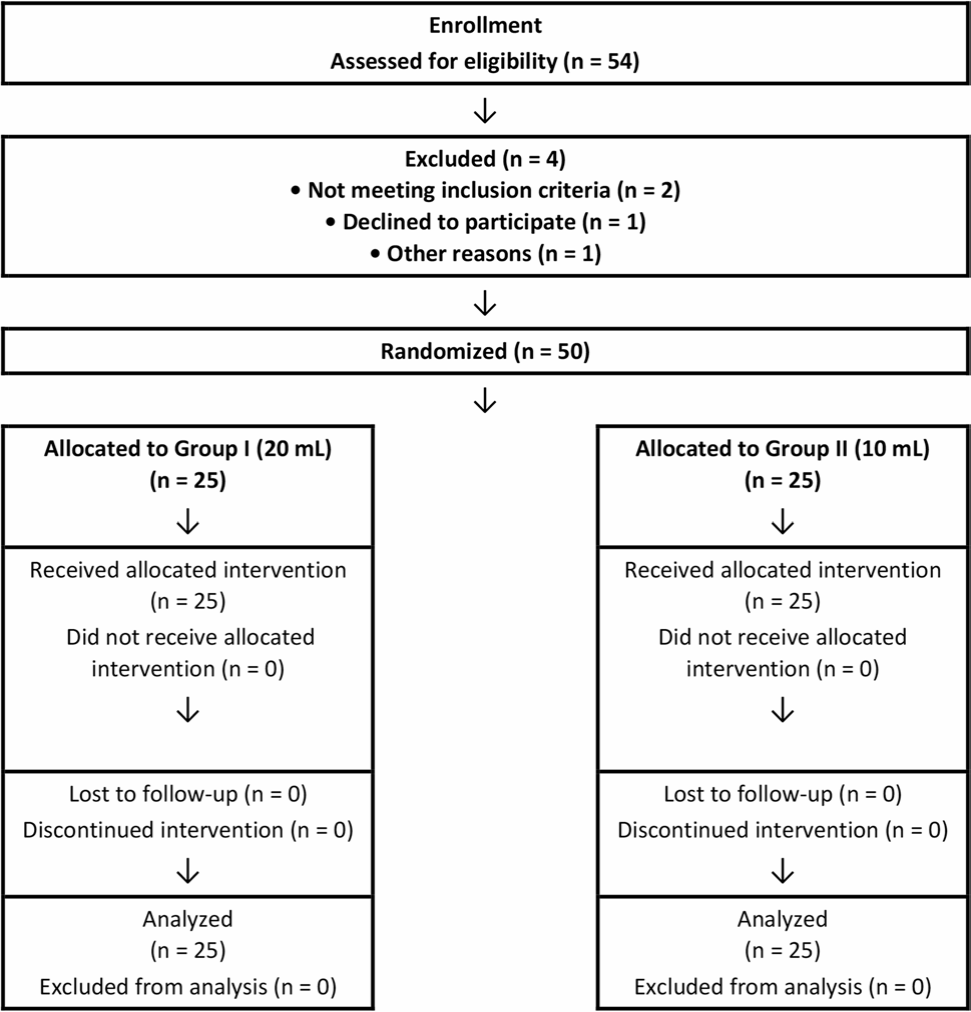
Flow diagram

This study adheres to the CONSORT guidelines for reporting randomized controlled trials, and a compliant flow diagram is provided as Fig. 1.

### Statistical analysis

All statistical analyses were performed using IBM SPSS Statistics for Windows, version 30.0 (IBM Corp., Armonk, NY, USA). Continuous variables were assessed for normality using the Shapiro–Wilk test. Normally distributed data are presented as mean ± standard deviation (SD), and non-normally distributed data as median [interquartile range (IQR)]. Categorical variables are presented as numbers and percentages.

Between-group comparisons were performed using the independent samples t-test for normally distributed variables and the Mann–Whitney U test for non-normally distributed variables. Categorical variables were compared using the chi-square or Fisher’s exact test, as appropriate.

For repeated-measures analyses, PCA morphine consumption was analyzed using repeated-measures ANOVA. NRS pain scores are ordinal and were non-normally distributed; therefore, repeated NRS measurements were analyzed using the Friedman test.

### Non-inferiority analysis

The primary outcome (1-hour resting NRS score) was analyzed using a non-inferiority framework. A non-inferiority margin of 1 NRS point was prespecified based on IMMPACT recommendations, which consider a 1-point difference clinically meaningful in postoperative pain trials.

Non-inferiority was evaluated using a one-sided significance level of 0.025, corresponding to a two-sided 95% confidence interval. The between-group difference was calculated as (10 mL – 20 mL). Non-inferiority was concluded if the lower bound of the two-sided 95% confidence interval remained above the predefined non-inferiority margin (Δ = −1).

During manuscript preparation, AI-assisted tools (Grammarly and Microsoft Word Editor) were used for language polishing only; no generative content, data analysis, or scientific interpretation was performed.

### Sample size calculation

Sample size was calculated a priori for a non-inferiority design based on the primary outcome (1-hour resting NRS score). Assuming a standard deviation of 1.0, a non-inferiority margin of 1 NRS point, a one-sided α of 0.025, and 80% power, a minimum of 44 patients was required. To compensate for potential dropouts, the total sample size was increased to 50 patients.

## Results

A total of 54 patients were assessed for eligibility, of whom 4 were excluded (2 did not meet the inclusion criteria, 1 declined to participate, and 1 was excluded for other reasons). Fifty patients were randomized, with 25 patients allocated to Group I (20 mL) and 25 patients to Group II (10 mL). All patients received the allocated intervention and were included in the final analysis (Fig. 1). Variables with non-normal distribution were summarized using median [IQR], where appropriate.

When the patients’ ages, genders, BMIs, and ASA scores were examined, no statistical difference was found between the groups. Similarly, no differences were observed in comorbidities such as hypertension, diabetes mellitus, cardiovascular system disease, chronic obstructive pulmonary disease, smoking, chronic renal failure, or malignancy. In addition, no statistical difference was found between the groups in terms of anaesthesia duration and surgery duration (Table 1).

**Table 1:** Demographic data and comorbidities

	Group I (*n* = 25)	Group II (*n* = 25)	*P*-value	Mean or Median differences with 95% confidence intervals
Age	68.1 ± 10.4	69.4 ± 8.4	0.636	-1.3 years (95% CI, -6.7 to 4.1)
Sex (Male / Female)	5 (20%) / 20 (80%)	6 (24%) / 19 (76%)	0.742	OR 0.79 (95% CI, 0.21 to 3.03)
Body Mass Index (kg/m²)	23.8 ± 3.0	24.8 ± 4.5	0.370	-1.0 kg/m² (95% CI, -3.2 to 1.2)
ASA Scores				-
ASA-I	1(4%)	1(4%)	0.367	
ASA-II	19(76%)	15(60%)		
ASA-III	5(20%)	8(32%)		
Chronic Obstructive Pulmonary Disease	1(4%)	0(0%)	1	OR 3.12 (95% CI, 0.12 to 80.40)
Smoking	8(32%)	10(40%)	0.561	OR 0.71 (95% CI, 0.22 to 2.25)
Chronic Renal Failure	0(0%)	1(4%)	1	OR 0.32 (95% CI, 0.01 to 8.25)
Presence of Malignancy	0(0%)	1(4%)	0.490	OR 0.32 (95% CI, 0.01 to 8.25)
Duration of Anaesthesia (minutes)	120.2 ± 11.2	127.7 ± 18.1	0.091	-7.5 min (95% CI, -16.1 to 1.1)
Duration of Surgery (minutes)	105.4 ± 12.0	111.6 ± 16.5	0.136	-6.2 min (95% CI, -14.4 to 2.0)

Data expressed as mean ± SD or number and percentage (%)

*ASA* American Society of Anesthesiologists physical status classification

When the patients’ postoperative rest (passive) NRS scores at 30 min, 1 h, 2 h, 4 h, 8 h, 16 h, and 24 h were evaluated, no statistical difference was found between the groups (Table 2). NRS scores were compared temporally in all patients with the Friedman test, and significantly higher pain scores were observed in the first 2 h postoperatively compared to the following hours. While median NRS scores were ≥ 2 in both groups in the first 2 h, median NRS scores were determined as ≤ 1 after the 4th hour. These high pain scores were unrelated to the groups. When NRS scores were evaluated in the 30th minute, 1st hour, 2nd hour, 4th hour, 8th hour, 16th hour and 24th hour during movement (active), no statistical difference was found between the groups (Table 3).

**Table 2:** NRS Scores at Postoperative Rest (Passive)

	Group I	Group II	*P*-value	Median difference (95% CI)
	(*n* = 25)	(*n* = 25)		
NRS 30. Minutes	4[3–4]	4[4–4]	0.893	0 (− 0.5 to 0.5)
NRS 1. Hour	3[3–4]	3[3–4]	0.390	0 (− 0.6 to 0.6)
NRS 2. Hours	2[2–2]	2[2–2]	0.400	0 (− 0.5 to 0.5)
NRS 4. Hours	1[1–2]	1[1–2]	0.902	0 (− 0.6 to 0.5)
NRS 8. Hours	1[1–1]	1[1–2]	0.295	0 (− 0.5 to 0.5)
NRS 16. Hours	0[0–1]	1[0–1]	0.097	0 (− 0.6 to 0.7)
NRS 24. Hours	0[0–0]	0[0–1]	0.130	0 (− 0.5 to 0.5)

Data are presented as median [interquartile range]

*NRS* Numeric Rating Scale

**Table 3:** Postoperative active NRS scores

	Group I	Group II	*P*-value	Median difference (95% CI)
	(*n* = 25)	(*n* = 25)		
NRS 30. Minutes	5[4–5]	5[4–5]	0.397	0 (− 0.4 to 0.5)
NRS 1. Hour	4[3–4]	4[4–5]	0.277	0 (− 0.3 to 0.6)
NRS 2. Hours	3[2–3]	3[2–3]	0.581	0 (− 0.5 to 0.4)
NRS 4. Hours	2[1–3]	2[2–3]	0.353	0 (− 0.5 to 0.7)
NRS 8. Hours	2[1–2]	2[1–2]	0.557	0 (− 0.5 to 0.5)
NRS 16. Hours	1[1–2]	1[1–2]	0.287	0 (− 0.4 to 0.6)
NRS 24. Hours	0[0–1]	1[0–1]	0.249	0 (− 0.4 to 0.6)

Data are presented as median [interquartile range]

*NRS* Numeric Rating Scale

NRS scores reported with movement were compared temporally in all patients with the Friedman test, and significantly higher pain scores were observed in the first 4 h postoperatively compared to the following hours. In the first 4 h, median NRS scores were ≥ 2 in both groups, while after the 4th hour, median NRS scores were ≤ 2. These high pain scores were unrelated to the groups.

No statistically significant difference was found between the groups in terms of postoperative NSAID use and the number of patients. Postoperative PCA consumption was examined in 3 different periods as 0–8 h, 8–16 h, 16–24 h, and the average morphine consumption in 0–8 h (Group I: 24.8 mg and Group II: 26.1 mg), the average value in 8–16 h (Group I: 18.6 mg and Group II: 18.8 mg) and the average value in 16–24 h (Group I: 13.6 mg and Group II: 12.3 mg) was calculated. No statistically significant difference was found in morphine consumption in the 3 time periods and the total amount over 24 h.

Repeated-measures ANOVA demonstrated a significant time effect on PCA morphine consumption (*p* < 0.001), indicating that morphine consumption progressively declined across postoperative time intervals in both groups. No significant group × time interaction was observed (*p* = 0.451), suggesting that the temporal pattern was similar between groups. Post-hoc analysis with Bonferroni correction found that 0–8 h morphine consumption with PCA was statistically significantly higher than 8–16 h and 16–24 h consumption. However, no statistically significant difference was found in terms of 8–16 h and 16–24 h PCA morphine consumption (Table 4).

**Table 4:** Postoperative analgesic requirements and PCA morphine consumption (mg)

	Group I (*n* = 25)	Group II (*n* = 25)	*P*-value	Effect estimate (95% CI)
NSAID Requirement	6(24%)	5(20%)	1	OR 1.26 (95% CI, 0.33 to 4.84)
PCA Morphine Consumption 0–8 h (mg)	24.8 ± 7.7	26.1 ± 8.8	0.567	MD 1.3 (− 3.3 to 5.9)
PCA Morphine Consumption 8–16 h (mg)	18.6 ± 7.0	18.8 ± 8.3	0.931	MD 0.2 (− 4.1 to 4.5)
PCA Morphine Consumption 16–24 h (mg)	13.6 ± 7.5	12.3 ± 6.9	0.546	MD − 1.3 (− 5.3 to 2.7)
Total PCA Morphine Consumption (mg)	56.7 ± 19.3	56.9 ± 19.1	0.972	MD 0.2 (− 10.4 to 10.8)

Data expressed as mean ± SD or number and percentage (%)

*NSAID* Nonsteroidal anti-inflammatory drug, *PCA* Patient-controlled analgesia, *MD* Mean difference, *OR* Odds ratio, *CI* Confidence interval

No statistically significant difference was found between the groups in terms of opioid-related side effects (nausea, vomiting, pruritus), quadriceps muscle weakness, patient satisfaction level, hospital stay, and nausea-vomiting scale (Table 5).

**Table 5:** Opioid-Related Side Effects and Postoperative Patient Outcomes

Nausea	Group I (*n* = 25)	Group II (*n* = 25)	*P*-value	Median differences with 95% confidence intervals
	9 (36%)	9 (36%)	1	0 (0 to 0)
Vomiting	3 (12%)	2 (8%)	1	0 (0 to 0)
Itching	5 (20%)	6 (24%)	1	0 (0 to 0)
Quadriceps Muscle Weakness	0 (0%)	0 (0%)	1	0 (0 to 0)
Patient Satisfaction Level	5[5–5]	5[5–5]	0.610	0 (-1 to 1)
Hospital Stay Length	2[2–2]	2[2–2]	0.766	0 (-1 to 1)
Nausea-Vomiting Scale	0[0–1]	0[0–1]	0.944	0 (-1 to 1)

Data expressed as median [interquartile range] or number and percentage (%)

**Fig. 2:**
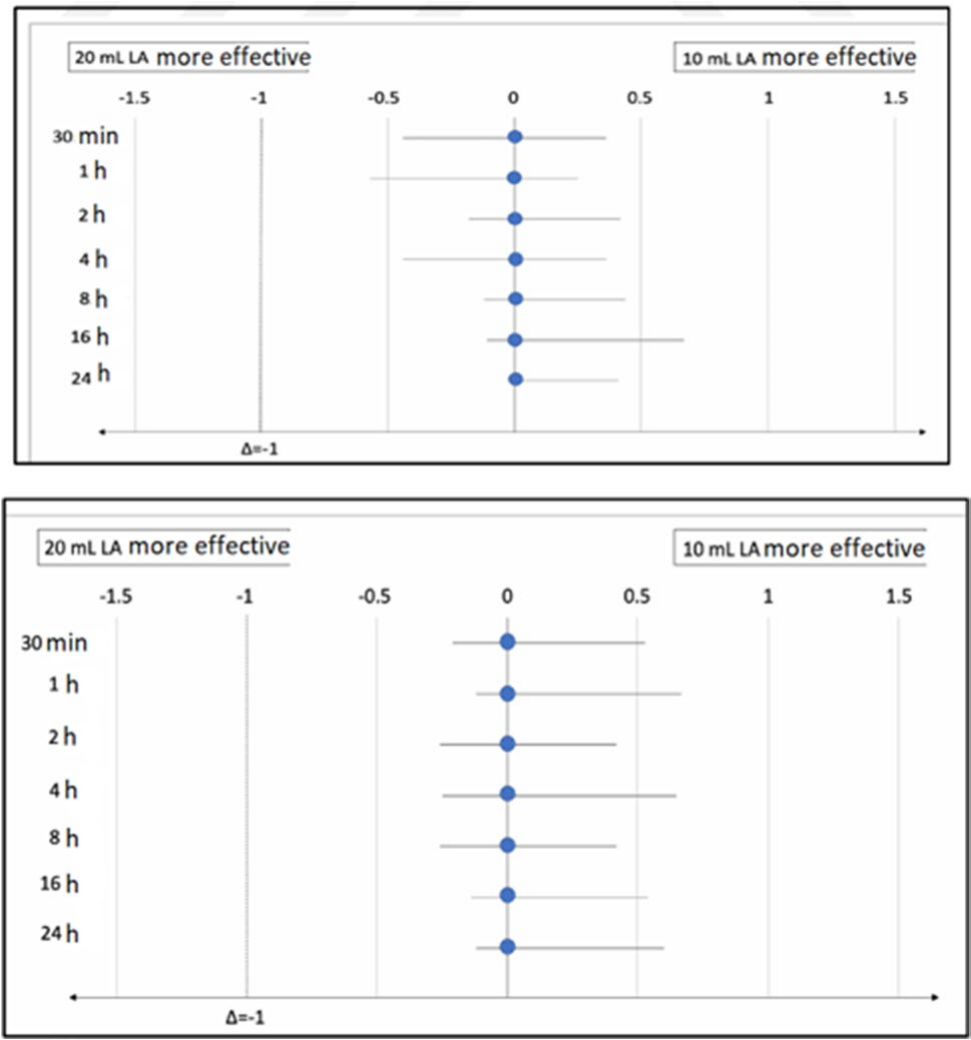
Non-inferiority analysis of postoperative NRS scores

For the primary outcome (1-hour resting NRS score), the between-group difference (10 mL − 20 mL) was 0 (95% CI − 0.6 to 0.6). Because the lower bound of the two-sided 95% confidence interval (− 0.6) remained above the predefined non-inferiority margin (Δ = −1), non-inferiority of the 10 mL PENG block was demonstrated. Similar findings were observed at other postoperative time points (Fig. 2).

## Discussion

In this randomized non-inferiority study, we demonstrated that a lower-volume PENG block using 10 mL of 0.25% bupivacaine provided postoperative analgesia comparable to that achieved with a 20 mL volume in patients undergoing total hip arthroplasty. Pain scores, rescue analgesic requirements, opioid consumption, motor function, and patient satisfaction did not differ significantly between groups during the first 24 postoperative hours. These findings suggest that effective pericapsular analgesia can be achieved without increasing local anesthetic volume, supporting the use of a lower-volume approach that may enhance the safety profile of the PENG block.

Previous studies evaluating the PENG block have used heterogeneous local anesthetic volumes, concentrations, and application timings, ranging from 10 mL to 30 mL and employing different agents and perioperative timings [9, 14–18]. In many of these studies, 20 mL has been considered a relatively low volume, although limited data suggest that lower volumes may be sufficient.

More recent randomized trials have yielded conflicting results: while Huang et al. found no clinically meaningful differences between 10 mL and 20 mL of ropivacaine [11], Wen et al. and Sood et al. reported superior analgesia with higher volumes, albeit with an increased risk of quadriceps weakness [10, 12]. Within this heterogeneous evidence base, our non-inferiority findings demonstrate that a 10 mL volume of 0.25% bupivacaine administered postoperatively provides analgesia comparable to 20 mL without increasing opioid consumption or motor weakness during the first 24 h after total hip arthroplasty.

From an anatomical perspective, the pericapsular region targeted by the PENG block represents a relatively confined fascial compartment in which the articular branches of the femoral, obturator, and accessory obturator nerves converge. Once this space is adequately saturated, further increases in local anesthetic volume may not proportionally enhance analgesic efficacy. Instead, higher volumes may increase the likelihood of spread to neighboring neural structures, potentially contributing to quadriceps weakness without additional analgesic benefit.

Multiple randomized trials and meta-analyses have consistently demonstrated that the PENG block provides effective postoperative analgesia with reduced opioid consumption and minimal motor impairment in patients undergoing hip surgery [8, 9, 19, 20]. Importantly, our findings extend this evidence by showing that similar analgesic and motor outcomes can be achieved with a lower local anesthetic volume, supporting a volume-sparing approach without compromising clinical efficacy.

Huda et al. [8] showed that PENG block prolonged 24-hour opioid consumption and the time to first rescue analgesia in patients undergoing hip surgery. Remily et al. [21] applied the PENG block to hip arthroplasty patients, and these patients had lower pain scores at 48 h, and their first opioid requirements were longer than those not applied. There was no difference between the study groups in terms of rescue analgesia requirements or the amount of analgesia administered in our study.

Yu et al. detected quadriceps weakness following a PENG block performed with a 20 mL local anesthetic volume [22], while Ahiskalioglu et al. reported quadriceps weakness in one patient after a 30 mL injection [23]. In contrast, no loss of quadriceps muscle strength was observed in any patient in the present study. This finding is clinically relevant, as preservation of quadriceps strength is essential for early mobilization and aligns with enhanced recovery after surgery (ERAS) principles. Consistent with our results, a meta-analysis demonstrated that the PENG block provides effective analgesia while preserving motor function and reducing opioid consumption compared with other regional techniques [8].

Patient-centered outcomes have also been favorably associated with the PENG block. Chung et al. reported increased patient satisfaction following PENG block application [20], although satisfaction scores were similar between groups in our study. Previous meta-analyses have shown that the PENG block is associated with reduced opioid consumption, less motor block, and improved range of motion, which may contribute to earlier postoperative ambulation in patients undergoing total hip arthroplasty [8, 19]. In the present study, length of hospital stay did not differ between groups. However, longer-term postoperative outcomes were not evaluated and should be addressed in future studies.

This study has several limitations that should be acknowledged. First, the single-center design and relatively small sample size may limit the generalizability of the findings. In addition, the study may have been underpowered to detect rare volume-related complications, such as subtle motor weakness or infrequent local anesthetic–related adverse events, which would require larger multicenter trials to be adequately assessed. Second, postoperative outcomes were assessed only within the first 24 h; therefore, longer-term analgesic effects, functional recovery, and the potential impact on chronic postoperative pain were not evaluated. Third, a single fixed concentration of bupivacaine (0.25%) was used, and the results may not be directly applicable to other concentrations or local anesthetic agents. Fourth, the administration of a standardized intraoperative morphine dose (0.1 mg/kg) may have attenuated early postoperative pain differences and opioid consumption between groups, potentially masking subtle volume-related effects. In addition, routine prophylactic antiemetics were not administered, and postoperative nausea and vomiting were managed with rescue therapy only, which may have influenced PONV-related outcomes. Finally, although outcome assessors were blinded, the anesthesiologist performing the PENG block was not blinded to group allocation due to the nature of volume preparation, introducing a potential risk of performance bias.

## Conclusion

This research investigated the effective and safe volume of the PENG block, used in hip surgeries, and found that the volume applied at 10 ml has an effect on postoperative pain as effectively and safely as the block applied at 20 ml.

## Supplementary Information

Supplementary Material 1.

## Data Availability

The data supporting this study’s findings are available on request from the corresponding author. The data are not publicly available due to privacy or ethical restrictions.

## Abbreviations

ASA: American Society of Anesthesiologists
BMI: Body Mass Index
EtCO₂: End-tidal carbon dioxide
LA: Local anesthetic
MAC: Minimum alveolar concentration
MRC: Medical Research Council
NRS: Numerical Rating Scale
NSAID: Non-steroidal anti-inflammatory drug
PCA: Patient-controlled analgesia
PACU: Post-anesthesia care unit
PENG: Pericapsular nerve group
PONV: Postoperative nausea and vomiting
RCT: Randomized controlled trial
SD: Standard deviation
SPSS: Statistical Package for the Social Sciences
THA: Total hip arthroplasty
